# Sensing in the Collaborative Internet of Things

**DOI:** 10.3390/s150306607

**Published:** 2015-03-19

**Authors:** João B. Borges Neto, Thiago H. Silva, Renato Martins Assunção, Raquel A. F. Mini, Antonio A. F. Loureiro

**Affiliations:** 1 Department of Computer Science, Universidade Federal de Minas Gerais, Belo Horizonte 31270-901, Brazil; E-Mails: thiagohs@dcc.ufmg.br (T.H.S.); assuncao@dcc.ufmg.br (R.M.A.); loureiro@dcc.ufmg.br (A.A.F.L.); 2 Department of Computing and Technology, Universidade Federal do Rio Grande do Norte, Caicó 59300-000, Brazil; 3 Department of Computer Science, Pontifícia Universidade Católica de Minas Gerais, Belo Horizonte 30535-901, Brazil; E-Mail: raquelmini@pucminas.br

**Keywords:** Internet of Things, sensor search and selection, Sensing Reliability

## Abstract

We are entering a new era of computing technology, the era of Internet of Things (IoT). An important element for this popularization is the large use of off-the-shelf sensors. Most of those sensors will be deployed by different owners, generally common users, creating what we call the Collaborative IoT. This collaborative IoT helps to increase considerably the amount and availability of collected data for different purposes, creating new interesting opportunities, but also several challenges. For example, it is very challenging to search for and select a desired sensor or a group of sensors when there is no description about the provided sensed data or when it is imprecise. Given that, in this work we characterize the properties of the sensed data in the Internet of Things, mainly the sensed data contributed by several sources, including sensors from common users. We conclude that, in order to safely use data available in the IoT, we need a filtering process to increase the data reliability. In this direction, we propose a new simple and powerful approach that helps to select reliable sensors. We tested our method for different types of sensed data, and the results reveal the effectiveness in the correct selection of sensor data.

## 1. Introduction

The ubiquitous computing paradigm [1] is increasingly becoming a reality. This is partially due to the revolution that we are now facing with the Internet of Things (IoT) [2,3]. An important element for this revolution is the popularization of a range of simple-to-use sensors, such as temperature, humidity, luminosity, and hundreds of others. IoT enables the proposition of more powerful services, the so-called IoT services [4], possibly combining different data sources. The main difference between an IoT service and a traditional Internet service is that the former is designed to interact with the physical world, by sensing and/or changing the state of physical entities. For instance, services that enable users to program basic behaviors (e.g., on/off) for their HVAC (heating, ventilating, and air conditioning) systems, and more powerful services for several areas, such as building, home automation and energy consumption, health care and transportation, security and public safety.

In this sense, IoT is much about exchanging and analyzing a large amount of unstructured and heterogeneous data, collected from different sources [2]. It is expected to be generated around 35 ZB (zettabyte) of data until 2020 [5], thanks to the increasing number of IoT devices. Unstructured and heterogeneous sensed data will have different characteristics and properties, which can be used to classify the collected data into two broad groups: data that has a reliable service to represent its “reference data”, and data that has not. Some common examples of the former group are temperature, pressure, humidity, wind speed and UV radiation that have their corresponding “reference” values available at different Web sites. Some common examples of the latter group are fine particulate matter, sound pressure level and concentrations of both organic compounds and metals in sediment and tissue that are not readily available. Notice that once IoT devices become more and more common, variables present in the latter group will start appearing in the former one. In this work, we only consider variables belonging to the first group that has reference values. Actually, the group with reference values represent variables that typically the sensor technology is widely available due to its advances/cost or importance for the society/particular users.

Regardless of the group, IoT devices are entities acting as providers and/or consumers of data related to the physical world, with different computing capabilities and resource limitations, ranging from small sensors to more powerful and complex systems. Notice that IoT encompasses different technologies such as wireless sensor networks, RFID tags and all sort of devices with embedded computing capability. However, our focus is on the data obtained from those entities, rather than on the physical systems aspects such as how the data was collected, processed and transmitted. In this work, we opted to adopt the term “sensor” for those devices/things, as it can also be found in the well-established literature of wireless sensor networks [6].

To deal properly with this massive data, a key feature is to have IoT services to support automated reasoning, which heavily depends on a standardized format and model for the collected data, *i.e.*, a semantic description of its content and metadata [2]. In a special case of IoT, which we call “Collaborative IoT”, most of these sensors will be deployed by different owners, generally common users, and for different purposes. In this scenario, the collaborative aspect comes from the fact that users make their data freely available by setting it as public. As a consequence, data privacy is not a concern, which might not be a problem for the kind of data at hand, whereas for other applications, security issues related to the IoT domain are typically a serious issue [7].

The Collaborative IoT helps to increase considerably the amount of data, but also brings several new challenges, being one of them the data quality. As mentioned, common users are responsible for these sensors, and, thus, it is not reasonable to expect they will provide the corresponding metadata, and guarantee both its data accuracy and availability. Another challenge is to search for and select a correct and reliable sensor or a group of sensors when there is no such description about the provided sensed data or when it is imprecise. Considering these aspects, the main research questions of this work are:

1. How reliable is the available sensed data in IoT and which aspects impact its accuracy?
2. Is it possible to combine sensed data from different sensors in order to improve the data quality?

In this work, we characterize the properties of real sensed data in the Internet of Things, potentially contributed by different sources, including sensors from general users, *i.e.*, the Collaborative IoT. We conclude that, in order to safely use data available in the IoT, it is necessary a filtering process to increase the data reliability. In this direction, we propose a new simple and powerful approach that helps to select reliable sensors, exploring collaborative filtering [8]. We tested our method for different types of sensed data and the results show that the proposed method is very effective to select reliable sensors, regardless if they are part of a controlled set of sensors or not.

The rest of this paper is organized as follows. In Section 2, we briefly discuss the main efforts in sensor search and selection strategies, including Internet services and IoT services, and discuss some available platforms for sensing in IoT, examining the quality assurance of those services. In Section 3, we investigate the properties of sensed data in the collaborative IoT and propose strategies to deal with its data uncertainties. Section 4 presents an experiment with real sensed data, in order to evaluate the feasibility and limits of our approach. Finally, in Section 5 we present our conclusions and future research directions.

## 2. Related Work

There are at least two aspects to consider when we study sensing in the IoT environments. The first one is a proper sensor search and selection strategy to ensure the desired sensors are found. The second one deals with the quality of these selected sensors, since the behavior of a system depends completely on the observations it infers about the environment, which is based on the sensed data.

### 2.1. Solutions for Sensor Search and Selection

The first aspect that may affect the quality of the sensing is how to correctly match the expected data to the actually collected one. The description of the services plays a key role to this, since the richer the service description is the better its matching will be [9] and, thus, it allows us to better interpret and understand the service itself [10]. To address this problem, the solutions should begin by a proper description of the service functionalities [2].

As established by Mian *et al.* [11], service description is an abstraction of a service's functionalities and characteristics. A simple and unambiguous method for describing services is to use the UUID (Universal Description, Discovery and Integration) [12]. However, even this method, that results in a practical unique service identifier, has no relation to the service itself, *i.e.*, it cannot capture the service details in order to help users to choose the service according to its specificities, nor the users will be able to compare two different services.

Zang *et al.* [13] point out that a proper service description is not enough to suggest the best service to the users' needs. Instead, to handle the characteristics of IoT services, it is indispensable that the consumer be able to perceive the current state of the service it will require. In other words, the service consumer should follow the principles of context awareness [14] and able to intelligently identify and autonomously adapt to service changes. Thus, in addition of being aware of the state of its users, an IoT-based system should also be able to take into account the dynamics of the IoT services they are based on.

To address this question, Perera *et al.* [15] propose an ontology-based context framework to allow a comprehensive sensor search and selection functionality that best suits the user requirements. Once the system knows the user requirements and priorities, it will be able to search for the appropriated sensors, rank them according to the described preferences and select the number of sensors the user defines.

On the other hand, the service discovery, which fully depends on the service description, still needs a special attention. Ververidis *et al.* [16] define service discovery as a process that enables network entities to advertise their services, query about services provided by other entities, select the most appropriate matched services and invoke them. The goal of that work is to facilitate the use of the IoT platform by automating the service operation and making a seamless integration between the physical and the informational worlds [17].

There are several proposals to deal with the service discovery challenge in the Internet domain. For the Web service discovery, the UDDI also provides a directory service where entities can register and search for services [18]. The most recent WS-Discovery solution [19] deals with the Web service discovery tasks. Other examples of service discovery are the Apache River [20], the advancement of the Sun's Jini, Universal Plug and Play (UPnP) protocol [21], Service Location Protocol (SLP) [22] and the Bluetooth Service Discovery Protocol [23]. However, in the IoT scenario, there are more challenges to solve than simply applying those strategies, as discussed above.

Wei *et al.* [17] also state that context-aware service discovery approaches should be used to provide, besides autonomous adaptation, the ability to deal with the uncertainties and temporal contexts of IoT. Rambold *et al.* [24] highlight that an autonomic service discovery approach should consider the possibility of monitoring a service and, in case it is not available (because of a service failure, for instance) or its description is outdated, to discover a service replacement. Rong *et al.* [9] present a survey and classification of the main discovery approaches for context-aware Web services.

### 2.2. Solutions for the Quality of Sensing in IoT

Besides the previously mentioned efforts about strategies for searching and selecting one or more sensors, considering specific contextual information from them, the current state of the art of a collaborative IoT environment is still far from providing complete information available. For instance, the most common pieces of information available from a sensor platform and middleware for an IoT application are the *sensor location*, *sensor type*, *keywords* and the data they generate. Some examples of these platforms are the Linked Sensor Middleware (LSM) [25], the Global Sensor Networks (GSN) [26], the Microsoft SensorMap [27] and the Xively platform [28].

To properly search for and select a desired sensor, the designer of an IoT system must consider those pieces of information and the collected data itself, including its quality. This means that, once a sensed data is given, it will be necessary to analyze its behavior and detect if this is the desired data, *i.e.*, if this data is similar to the expected data generated by a correct sensor (reference value). This can be useful for both searching for a desired sensor and determining which sensor to select when a set of sensors is available.

In general, the sensed data will be used in context-aware systems [14], and, thus, the quality of the sensed data can be compared to the Quality of Context (QoC) concept. Buchholz *et al.* [29] define QoC as any information that describes the quality of information used as context information. They also establish the differences between QoC and QoS (Quality of Service), which refers to any information that describes how well a service performs, and QoD (Quality of Device), which means any information about the devices' technical properties and capabilities. According to those definitions, they propose the following QoC parameters to measure the quality of a context [29]: *precision*, *probability of correctness*, *trustworthiness*, *resolution* and *up-to-dateness*.

The *precision* of the sensed data is related to how the sensed data reflects the current reality of the physical phenomenon. *Probability of correctness* denotes the probability that a given data is correct. *Trustworthiness* is similar to the probability of correctness, but it is used to rate the quality of the entity which generated the data. *Resolution* denotes the information granularity, and *up-to-dateness* describes the age of the data.

Li *et al.* [30] extend the QoC definition for pervasive environments. They investigate the challenges in providing data quality in these environments and propose three metrics to quantitatively observe the quality of these data and their data sources: *currency*, *availability* and *validity*. *Currency* is related to the up-to-dateness metric above, but it represents the temporal utility of the data, from the moment it is created until is useless. The *availability* is more concerned about the capability of an entity to generate data. Finally, the *validity* is defined by a set of rules that can be used to validate the sensed data according to the previous knowledge about the expected properties of the data.

Despite the fact that all these metrics asses data quality, the current collaborative IoT environments suffers from a lack of information about their services, which can impact the correct analysis of their data and sources. In general, there is neither a precise description about the data a sensor generates nor the functionalities they provide, nor a metadata about the state of these sensors. Furthermore, we can not expect to have a different scenario in the short term since this would probably call for a standardization effort in that case. Thus, it is necessary to investigate this current scenario and analyze possible solutions to improve the reliability of IoT services to be deployed.

In that direction, Sicari *et al.* [31] propose an architecture to ensure both the security and the quality of IoT data. The main aspect in that work related to our discussion is that the collected data is first processed and analyzed to extract relevant information about it (quantitatively) and from its sources. The resulting information becomes its metadata.

In our work, we consider a previous step before employing the sensed data, since in the collaborative IoT we can not rely on the information obtained from sensors and their services. More specifically, we study the worst case scenario, where no information about the data exists at all, and discuss a method to select the appropriate data to be used. We perform our analysis and discuss our assumptions based on properties and behavior we observe from the real data itself.

## 3. Characterizing the Sensed Data

The study of the properties of the sensed data in the collaborative IoT may be very useful to define strategies to deal with sensor data uncertainties. To investigate that, we collected and analyzed public data from real sensors deployed in a city-wide area. In this section we study the properties of that sensed data and present two algorithms to improve its reliability, establishing a proper sensor selection and refinement of the sensed data.

### 3.1. Setup

In the following analysis, we consider two datasets, both collected from the region around London, UK, extracted from a collaborative IoT. The first dataset includes only temperature sensors, *i.e.*, sensors in which the temperature tag was included in their descriptions. Note that this procedure may not consider some temperature sensors whose description does not include this tag. The second dataset includes all collected sensors, without any sort of filtering. We were able to collect data for temperature, humidity, pressure, wind speed, and more specific sensors, such as binary sensors that indicate the turn on/off of lights and watts consumption.

Those datasets will allow us to asses the behavior of the sensed data in a controlled environment and, then, relax this criterion to include different types of data. The parameters of these collections are detailed in Table 1. We also collected reference values from conventional weather forecast services. This reference was used to select appropriate temperature sensors (Dataset 1). For this particular case, we were interested in outdoor temperature data. To select those sensors, we compared data generated by sensors and data generated by the weather forecast service (reference service) computing the correlation coefficient among them.

The selection of a reference service concerns the reliability we should have on the data generated by that particular source. Obviously, to determine how trustworthy a service is, it is necessary a previous knowledge about both the sensing phenomenon itself and the corresponding reference values. For our study, it is easy to find a reliable reference service for our data type. Moreover, for other data types, we may use the reputation of the related service to asses its reliability. In this sense, some criteria already discussed here can be used in this assessment, such as the precision of data generated, probability of correctness, trustworthiness and availability [14].

**Figure 1.**
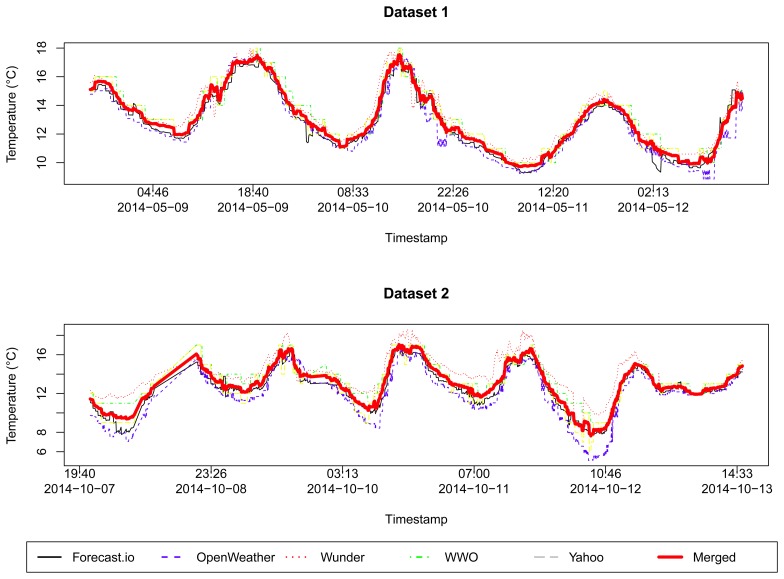
Comparison between five possible reference services and the average merged sample, for the two datasets.

We considered the following five options of weather forecast services as our reference: Forecast.io [32], Open Weather [33], Weather Underground [34], World Weather Online [35] and Yahoo Weather [36]. As shown in Figure 1, all weather forecast services were similar in the period of the analysis. We have chosen the Forecast.io service as our reference data, since it is close to the average merged sample, with a mean squared error (MSE) of 0.0685161 for Dataset 1, and 0.1571421 for Dataset 2.

Notice that IoT scenarios will comprise a massive amount of unstructured and heterogeneous data, leading to different data classes, which have their own characteristics, boundaries, trends and patterns. As already discussed, in this work, we only consider variables belonging to the group that has reference values. The process of suggesting a reference value for variables that do not have a reference data service promptly available is still an open issue and will be addressed in the future work.

In this work, we analyze the data class with a reference service by taking as our case study a single and controlled variable: weather data. This data class has been studied at a great extent in the literature of sensor networks. Furthermore, it exemplifies the sort of sensing data we can find several reliable weather forecast services to represent our reference data. For different and more specific data classes, it is not possible yet to obtain a reference as simple as this. For those cases, we need a better understanding about the corresponding classes and their features.

**Figure 2.**
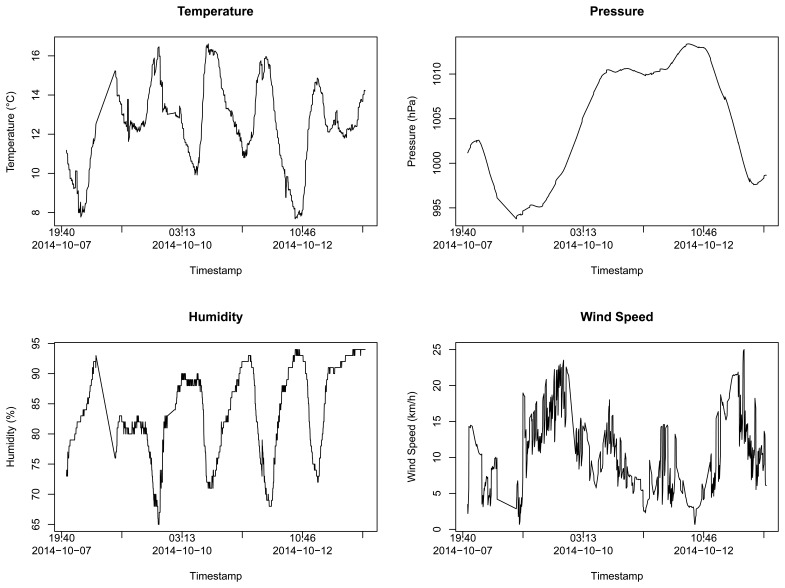
Reference data samples for different types of data temperature, pressure, humidity and wind speed (Dataset 2).

We also collected four different types of data from Forecast.io weather forecast service: temperature, pressure, humidity and wind speed. Figure 2 illustrates the time series behavior of the obtained samples.

Based on these characterizations, we designed algorithms for creating sensing services in the current collaborative IoT environments, establishing two directions:

A) Sensor search and selection, and
B) Sensor refinement.

### 3.2. Sensor Search and Selection

To search for a real sensor data, we rely on the public available data from Xively [28], a popular collaborative IoT platform. Through that Web service, users can make available their sensor readings to the cloud, directly from the embedded devices, collect data using a common API, and read and manage data from a remote application or a Web browser. In the Xively platform, the sensor data can be grouped into blocks called feeds, each one having the following attributes: feed id, tags, status, description, location (e.g., name of city or coordinates) and data streams. A data stream is the sensed data obtained from a sensor and is described by its data stream id, tags, current value, min and max values and the unit of the data generated. In some cases, not all of those pieces of information are filled by the sensor owners or, when they are provided, they are not precise.

Searching for the desired sensors using the Xively REST API is simple and can be accomplished through a Web browser, such as the following example:
http://api.xively.com/v2/feeds.json?  key=XIVELY_API_KEY&lat=51.53&lon=−0.10&  q=temperature&distance=50&status=live

In this example, we are searching for sensors located in a range of 50 km from the coordinates of London, UK, that have in their description the tag “temperature” and in which the status is live, meaning the sensor is actually generating data.

As we can see, the Xively query above is very simple and only contains a tag and coordinates. This means that the sensed data can be different from what we are looking for. Besides, the result does not include context information about the data itself, which can lead to a wrong sensor selection, impacting the quality of the sensed data.

**Figure 3.**
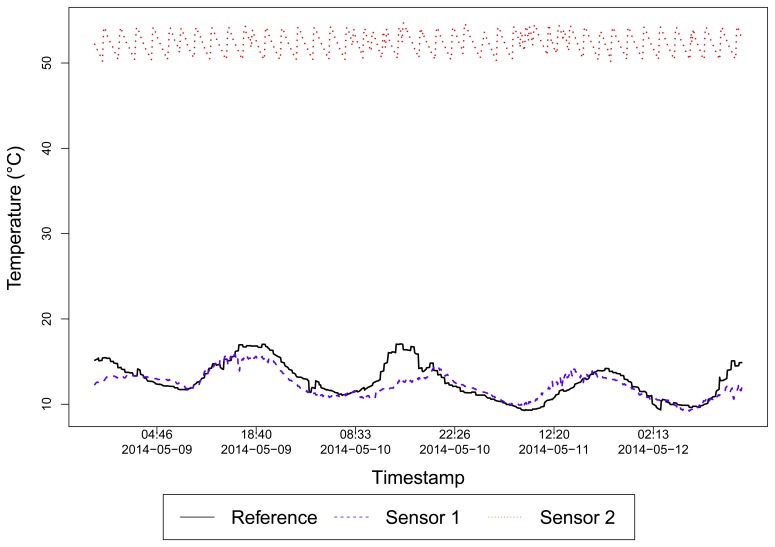
Different sensor readings for the “temperature” tag, but with different meanings (Dataset 1).

For example, Figure 3 shows different sensor data collected for temperature. While the blue dashed line (Sensor 1) means a sensor reading for an outdoor temperature, the red dotted line (Sensor 2) corresponds to a sensor reading for the temperature of a water tank. For this specific case, the data owner filled the description of the temperature data, but this is not the general case. The black solid line represents our reference data. This exemplifies the need for a specific sensor selection strategy for this kind of sensed data.

In our case, we want to select sensors measuring temperature, located at a given range from a given coordinate, but that their temperature corresponds to similar conditions of the outdoor environment. Since there is no way to specify this requirement in the search parameters, the application must infer, according to the data value, whether it could be generated by a reliable sensor or not.

It is clear the difference between reliable and unreliable sensor data. A reliable sensor generates data more correlated to the reference data, and can be a trusted source of the temperature, in our example. Thus, if the reference corresponds to the temperature of the outdoor environment, a trusted sensor data will be the one that is somehow influenced by the environment temperature variation. This influence is measured by behavior similarities among the two sensed data.

For our first analysis, we consider the statistic measure of the Pearson's sample correlation coefficient (*r*) between the reference data and the collected data from the sensors. The formula for *r* is described in Equation (1),

(1) $$r=\frac{\sum_{i=1}^{n}\left(X_{i}-\bar{X}\right)\left(Y_{i}-\bar{Y}\right)}{\sqrt{\sum_{i=1}^{n}{\left(X_{i}-\bar{X}\right)}^{2}\sqrt{\sum_{i=1}^{n}{\left(Y_{i}-\bar{Y}\right)}^{2}}}}$$

where *X_i_* is the *i*th value of the reference data, *Y_i_* is the *i*th value of the sensor data to compare, and *X̄* and *Ȳ* are their sample mean, respectively. The value of r can vary between [−1,1], and the more correlated two samples are, the closer to one is |*r*|.

**Figure 4.**
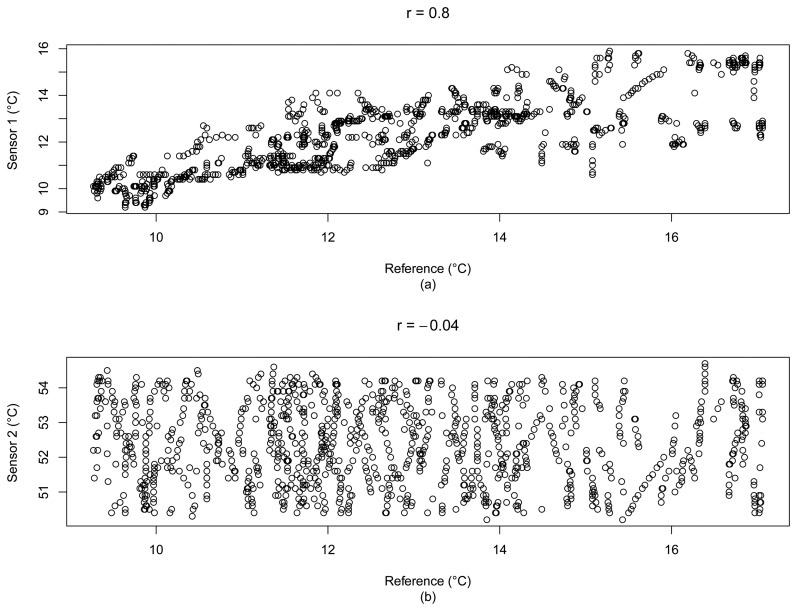
Scatter plot between the reference data (*x*-axis) and (a) the correlated Sensor 1; and (b) the uncorrelated Sensor 2 (Dataset 1).

For the example of Figure 3, the correlation coefficient between the outdoor Sensor 1 and the reference data is *r* = 0.8, and for the particular water tank temperature Sensor 2, *r* = −0.04, which can indicate that this is a good metric to filter trusted sensors in a given set. Figure 4 depicts a scatter plot that summarizes the correlations between the reference data (*x*-axis) and the cited sensors.

**Figure 5.**
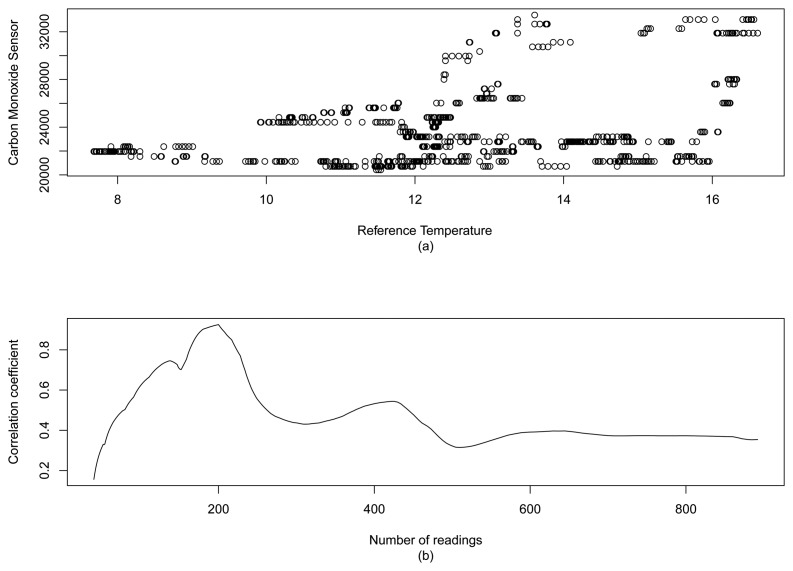
(a) Scatter plot between a temperature and a carbon monoxide sensor; and (b) the analysis of its correlation coefficient when the number of samples increases (Dataset 2).

However, the correlation coefficient can not be the only parameter to indicate whether a sensor is reliable or not. For example, consider Figure 5, which represents the analysis of the correlation coefficient between two naturally uncorrelated data samples, our temperature reference data and a carbon monoxide (CO) sensor, from Dataset 2. Since the correlation coefficient *r* does not consider the scale of the compared data (see Equation (1)), even if the magnitude of the samples are very apart, the correct values of *r* will depend on the number of considered samples. For the case of Figure 5b, we can observe the increasing of *r* up to 0.9255456, when we have only 200 samples. After that point, the value of *r* tends to decrease, and with 892 samples we have *r* = 0.3539141. Thus, if we consider a sensor to be reliable, in our case we would need a correlation value *r* = 0.8. To assume that the sensor is unreliable, we would need at least 224 samples.

Notice that we can not depend on the amount of samples we have, as stated before in Section 2.2, and thus, we need to include in the sensor selection strategy more knowledge about the data we are dealing with. As proposed by Li *et al.* [30], we also consider a set of validity rules 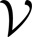 = {*υ_i_*,…,*υ_l_*} sensors must satisfy to be considered reliable. The data validity is a metric based on the estimation that the observation, according to the specificities of each problem, does not deviate from an acceptable range, in comparison to a known and expected behavior for that data. For example, in London, UK, the average maximum temperature between June and September is around 23 °C whereas the minimum is 11 °C.


**Algorithm 1:** Sensor Selection
**Input**: **S***_n_*_×_*_m_* matrix of time series for *n* sensors with *m* samples each, a vector **r** = {*r*_1_,…, *r_m_*} of a referencetime series, a correlation coefficient limit *ρ* ∈ [—1,1], and a set of validity rules 

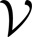
 = {*υ*_1_,…, *υ_l_*}.**Output**: A set 

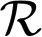
 of reliable sensors.*n* ← rows(**S**);*m* ← cols(**S**);

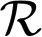
 ← ∅;// 
For each sensor entry in the time series matrix
**S.****for**
*i* ← 1 **to**
*n*
**do** // 
Adjust the valid readings among the reference and the sensor.  s* ← { *s_i,j_ (s_i,j_* ≠ λ) and (*r_j_* ≠ λ), 1 ≤ *j* ≤ *m* };  r* ← { *r_j_* (*s_i,j_* ≠ λ) and (*r_j_* ≠ λ),1 ≤ *j* ≤ *m* }; // 
Compute the correlation coefficient between
s* 
and
r*.  ρ* ← cor(s*, r*); // 
The correlation satisfies the given limit?  **if**
ρ* ≥ *ρ*;  **then**  // 
The sensor entry satisfies all validity rules?  **if**
$\wedge_{k=1}^{l}\upsilon_{k}\left(\text{s}*\right)$;  **then**    

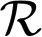
 ← 

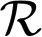
 ∪ *i*;  **end** **end****end****return 

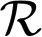
**


Algorithm 1 summarizes this sensor selection strategy considering the aforementioned issues. It is important to emphasize that the readings must be adjusted, since, in a given time, a sensor might fail sending its readings, and this gap can be a problem in the computation. In the algorithm, we adjust this by computing new vectors s* and r* in which their values correspond to the entries that occur in both sensor and reference readings, respectively, *i.e.*, there are no null values (λ) for both vectors.

### 3.3. Sensor Refinement

**Figure 6.**
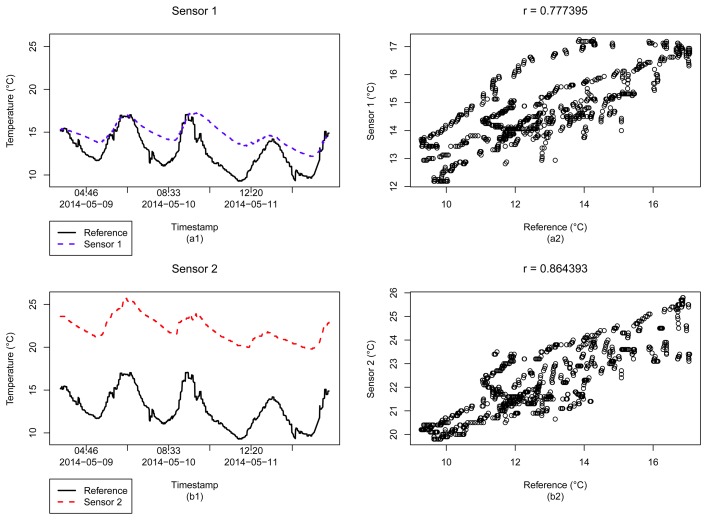
Time series (**a1**) and scatter plot (**a2**) analysis for a *r* = 0.777395 related sensor, and time series (**b1**) and scatter plot (**b2**) analysis for a *r* = 0.864393 sensor (Dataset 1).

The correlation coefficient and the validity rules seem to be reasonable metrics to be used as a first strategy for sensor selection, to filter the sensors that best fit our purposes. However, in order to safely use all data from these sensors, we still need to consider some issues. For instance, consider the example of Figure 6, from Dataset 1, which illustrates the time series and scatter plots between two sensed data and a reference data. Even with a relatively high correlation between the sensors and the reference, *r* = 0.777395 for Sensor 1 and *r* = 0.864393 for Sensor 2, the reading values given by the sensors are systematically higher than that provided by the reference. For the case of Sensor 1, apparently the temperature values follow the increase of the environment temperature, but present a slower response to its decrease. However, for Sensor 2 readings, we can see that, while the measured temperature values are related to the reference, *i.e.*, there is an influence of the environment temperature in this sensor, all its values are an order of magnitude higher than the reference values.

In Figure 6, when we analyze the temperature measured by Sensor 2, we can argue that the given metrics can not ensure the reliability of this sensor data. Note that the correlation coefficient (≈0.8 for this case) can represent an influence of the environment temperature on the sensor, and can be considered acceptable for the validity rules at the period of the reading. However, it gives no more information about the correctness of the data, and how reliable the sensor is. We can not make sure whether the temperature is really higher at that location or the data is coming from an indoor sensor, which would explain that value.

**Figure 7.**
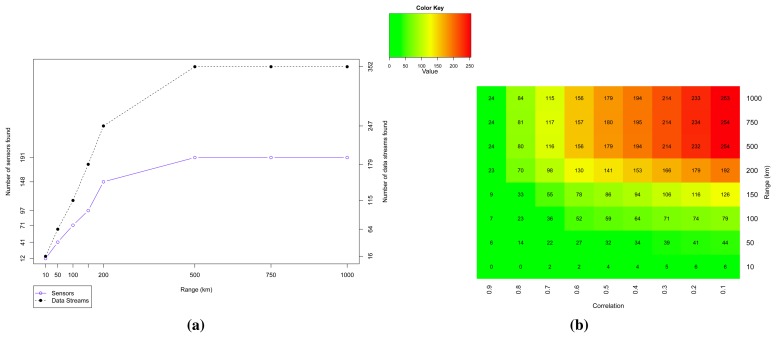
Analysis of the number of sensors found in Dataset 1. (**a**) Number of sensors and their data streams found for different sensor search ranges (Dataset 1); (**b**) Heat map for the number of data streams found when varying the range of the desired location area and the correlation coefficient limit (Dataset 1).

On the other hand, when we look at the bigger picture, we can observe some interesting patterns that can be used to handle this uncertainty. Figure 7a shows the sensors and their data streams found in Dataset 1. As we increase the range (distance in kilometers from the central coordinate point), the number of available sensors (feeds) and their data streams also increase. On average, a sensor provides approximately two data streams (1.84). Applying the sample correlation coefficient *r* as a metric to filter the considered trusted data, we have the results showed in the heat map of Figure 7b, which represents the selected data streams when varying the range and the correlation coefficient limit *l*. From the set of all data streams in a given range, we selected those which give *r* ≥ *l* in comparison with the reference sensor data.

**Figure 8.**
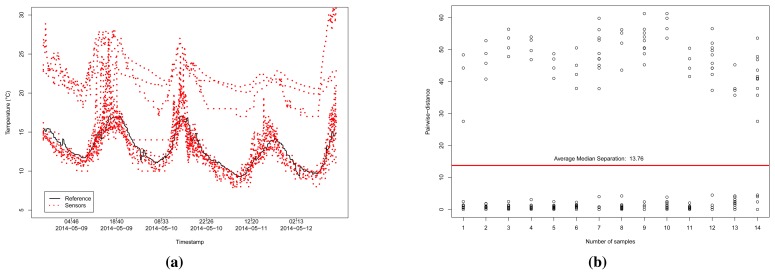
Analysis of the time-series and pairwise-distances between 14 reliable sensors with *r* ≥ 0.8 to the reference temperature data (Dataset 1). (**a**) Time-series analysis of those 14 reliable sensors; (**b**) Pairwise-distance between the time series of the reliable sensors.

If we take those sensors considered reliable within a range of 50 km and the correlation coefficient *r* ≥ 0.8, we have 14 sensors to handle. Figure 8a illustrates a time series analysis of those sensors. Given the 14 reliable sensors, which we assumed to be influenced by the environment temperature, ten of them are close to the reference data, and four are dislocated by a given value Δ*_i_*, for each sensor *i*, considering the value of the reference sensor.

Given a set 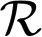 = {*r*_1_, *r*_2_,…, *r_n_*} of reliable sensors, let 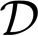 ⊆ 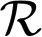 be the subset of sensors that are dislocated from the reference values, which we assume to be the correct temperature value for that region at a given time *t*. Let us assume to be constant the amount Δ*_i_* a sensor *r_i_* is dislocated from the reference sensor throughout time *t*. Thus, the time series *y_i,t_* for each sensor *r_i_* can be defined as

(2) $$y_{i,t}=\theta_{t}+\Delta_{i}I_{\left[r_{i}\in D\right]}+{ɛ}_{t}$$

where *θ_t_* is the reference sensor value at time *t*, *ε_t_* ∼ *N*(0, *σ*^2^) is a Gaussian random variable that represents an i.i.d. (independent and identically distributed) error with expected value 0 and variance *σ*^2^ at *t*, and *I*_[_*_r_i__*_∈_**_
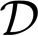
_**_]_ is a binary random variable, where *I*_[_*_r_i__*_∈_**_
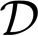
_**_]_ = 1 if the sensor *r_i_* belongs to the set 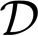, or *I*_[_*_r_i__*_∈_**_
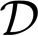
_**_]_ = 0, otherwise.

Based on that, we can define an estimation *θ̂_t_* of the value of the reference *θ_t_*, using Equation (2), as

(3) $${\hat{\theta }}_{t}=\frac{1}{n}\sum_{k=1}^{n}\left(y_{i,t}-{\hat{\Delta }}_{i}{\hat{I}}_{\left[r_{i}\in D\right]}\right)$$

To estimate *Î*_[_*_r_i__*_∈ 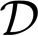]_, we compute a pairwise-distance matrix **P**_*n*×*n*_ for the time series of the correlated sensors, where each element *p_i,j_*1 ≤ *i*,*j* ≤ *n*,*i* ≠ *j*, corresponds to the average squared distance between the two time series of sensors *r_i_* and *r_j_*, and is denoted by

(4) $$p_{i,j}=p_{j,i}=\frac{1}{T}\sum_{t=1}^{T}{\left(y_{i,t}-y_{j,t}\right)}^{2}$$

where *T* is the final time *t* in the time series.

Figure 8b illustrates the pairwise-distance between those 14 sensors, according to the computed values of **P**. We can see that most of the correlated samples have a small distance among themselves, and a higher distance for the dislocated sensors. Thus, sensors that have the median pairwise-distance above the average median of them, the red straight line in Figure 8b, can be considered to belonging to 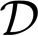. In this case, 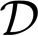 = {*r*_7_, *r*_9_, *r*_12_, *r*_14_}.

The estimated value Δ̂*_i_* for each sensor *r_i_* dislocated from the non-dislocated sensors can be denoted as the median of deviations from all the pairwise-distances between this sensor and the others, *i.e.*, for each row *i* of **P**, we can compute Δ̂*_i_* as follows

(5) $${\hat{\Delta }}_{i}=\text{median}\left(p_{i,j}-\text{median}\left(p_{i,j}\right)\right),1\leq j\leq n.$$

This equation is a slight variation of the median absolute deviation, without the absolute value computation, to adjust the correct value, positive or negative. For our example, Δ̂_7_ = 11.7309425, Δ̂_9_ = 9.2068333, Δ̂_12_ = 9.8445421, and Δ̂_14_ = 11.8916635.

Algorithm 2 describes the necessary steps to compute the set 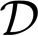 of dislocated sensors and their dislocation values Δ*_i_*, ∀*_i_* ∈ 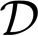.

Considering the points mentioned above, we can argue that, for a sufficient large number of sensors, if we observe a pattern between the correlated sensors, it is possible to estimate the correct signal of the temperature at a given region. This is accomplished by a collaborative filtering strategy [8], assuming that most of the sensors in such a region have a higher tendency to be close to the correct temperature value. Also, based on this assumption, we can estimate the deviation Δ*_i_* of sensors that are a magnitude apart from the majority, to improve the quality of the sensed data.

An important point to discuss regarding the benefits of the proposed strategy is about the security concerns of the obtained data quality in the Collaborative IoT Coen-Porisini *et al.* [37] present a detailed study of the problem of a malicious sensor generating erroneous data, and, here, we emphasize two malicious behaviors: (i) data is modified by a constant factor; and (ii) data is modified by the addition of a random error, a noise.


**Algorithm 2:** Sensor Refinement
**Input**: **S***_n_*_x_*_m_* matrix of time series for *n* sensors with *m* samples each, and a set 

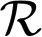
 = {*r*_1_, *r*_2_,…, *r_n_*} of reliable sensors.**Output**: Set 

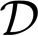
 of dislocated sensors and vector Δ of their dislocations from the majority.

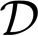
←∅Δ ← {Δ*_i_* = 0 | 1 ≤ *i* ≤ *n*}**m** ← {*m_i_* = 0 | 1 ≤ *i* ≤ *n*}**P***_n_*_×_*_n_* ← {*p_i,j_* = 0 | 1 ≤ *i,j* ≤ *n*}// 
Compute the average squared pairwise-distance between each two sensors.**for**
*i* ← 1 **to**
*n* − 1 **do** **for**
*j* ← *i* + 1 **to**
*n*
**do**  // 
Adjust the valid readings among the reliable sensors.  **u** ← { *s_r_i_,k_* (*s_r_i_,k_* ≠ λ) and (*s_r_j_,k_* ≠ λ), 1 ≤ *k* ≤ *m* };  **v** ← { *s_r_j_,k_* (*s_r_i_,k_* ≠ λ) and (*s_r_j_,k_* ≠ λ), 1 ≤ *k* ≤ *m* };  *p_i,j_* ← *p_j,i_* ← mean((**u** − **v**)^2^) **end****end**// 
Compute the median pairwise-distance for each sensor.**for**
*i* ← 1 **to**
*n*
**do** *m_i_* ← median({*p_i,j_*|1 ≤ *j* ≤ *n*})**end**// 
The limit to consider a sensor dislocated.*l* ← mean(**m**)// 
The dislocated sensors.

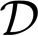
 ← {*i* | *m_i_* > *l*}// 
Compute the dislocation Δ*_i_*
for each reliable sensor.**for**
*i* ← 1 **to**
*n*
**do** Δ*_i_* ← median(*p_i,j_* − *m_i_*)**end****return** (

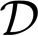
, Δ)


In the first behavior, the sensor readings are apart from the correct data values by a constant. In this case, the data signal still holds a reasonable correlation with the reference signal, being, thus, selected as reliable. After the refinement step, their values will be corrected according to the majority of sensors, by the collaborative filtering strategy. For the second case, when the data is modified by a random noise, it is presumable that, according to the magnitude of the noise increments, this signal will be considered unreliable and invalidated, and, thus, it will not be used. This occurs because it will not be correlated enough with the reference data, according to the correlation limit established.

## 4. Evaluation of Our Approaches

In the first experiment we use Dataset 1, described in Section 3, which contains only temperature data shared in the popular Xively collaborative IoT platform. We apply Algorithm 1 to search and select the most reliable sensors in this dataset.

The parameters passed to Algorithm 1 are the matrix of all sensors found in a range of 100 km from the coordinates of London, UK (see Table 1 for details), the Forecast.io weather service as the temperature reference data, and the correlation limit of *r* = 0.8. The validity rules for the class of temperature data were arbitrarily defined, based on a empirical knowledge of the data behavior, according to the following restrictions:

- The average temperature of the sampled data must be bounded by a factor of 2 from the average temperature of the reference data, *i.e.*, $\frac{1}{2}m_{\text{ref}}\leq m_{\text{smp}}\leq 2m_{\text{ref}}$, where *m_ref_* and *m_smp_* are the average temperature values for the reference data and the sampled data, respectively;
- The maximum temperature value of the sample max_smp_ can not be above the bound of 4 times the average reference temperature, max*_s_ mp* ≤ 4*m_ref_*; and
- The minimum temperature value of the sample min*_smp_* is lower bounded by a factor of 4 from $m_{\text{ref}},\min_{\text{smp}}\geq \frac{1}{4}m_{\text{ref}}$.

**Figure 9.**
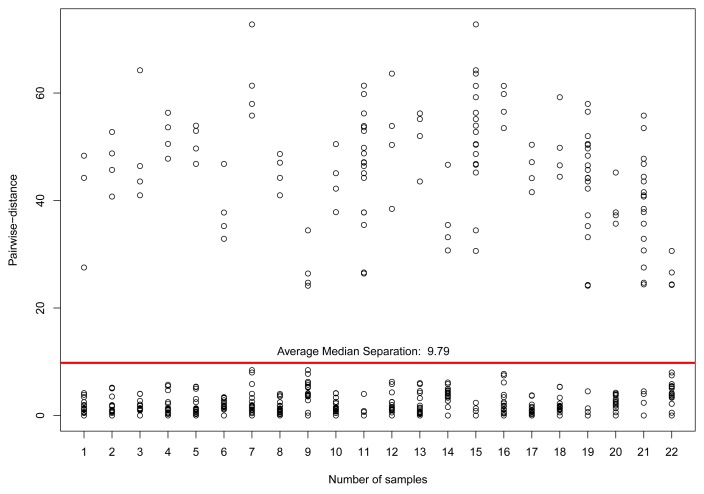
Pairwise-distances for temperature sensors in a range of 100 km from the coordinates of London, UK, in Dataset 1, considering only sensors with correlation *r* ≥ 0.8.

Figure 9 illustrates the pairwise-distance of the 22 selected sensors for this computation. After selecting those sensors, which we assume to be reliable, we compute the dislocation values Δ*_i_* for each sensor *i* applying Algorithm 2.

To validate our assumption that the combination of the readings for most of the reliable sensors converge to a correct value of the measured data, *i.e.*, the reference temperature data, we estimate the temperature *θ̂*.

**Figure 10.**
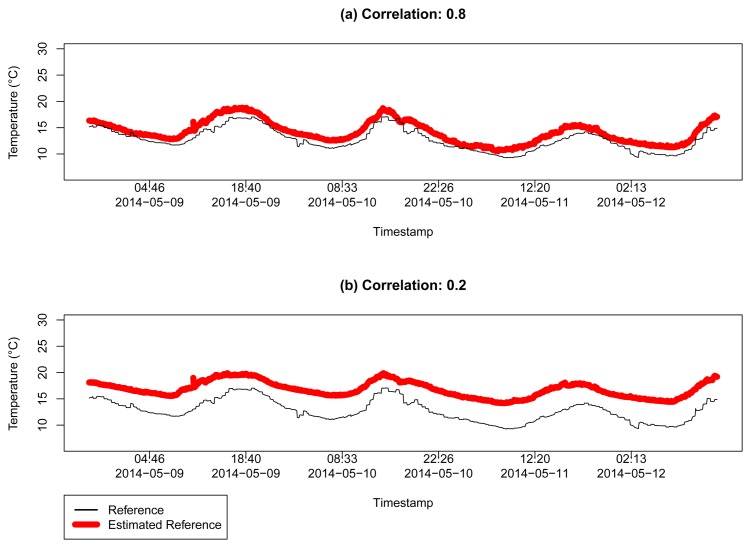
Estimation of the temperature *θ̂* for two different correlation limits in a range of 100 km from the coordinates of London, UK, in Dataset 1: (**a**) for sensors with correlation *r* ≥ 0.8 with MSE = 2.244199; and (**b**) for sensors with correlation *r* ≥ 0.2 with MSE = 16.85198.

Figure 10 shows the estimation of the reference data, based on Equation (3). Figure 10a shows the sensors that are 0.8 positive correlated with this reference, resulting in a MSE = 2.244199. Figure 10b shows the same estimation process, but relaxing the correlation criterion, considering all sensors with correlation coefficient *r* > 0.2 to the reference sensor, resulting in a MSE = 16.85198. As we can see, the more correlated the sensors are with the reference, the better the capacity of estimating *θ̂* is. Figure 10 also shows that the estimation error increases as we consider more unreliable sensors.

We did the same analysis for Dataset 2, but considering a broader range of data classes. In this case, the goal is to select a desired group of sensors from a set of heterogeneous classes of sensors. In this work, we consider selecting reliable sensors for temperature, pressure, humidity, and wind speed.

**Figure 11.**
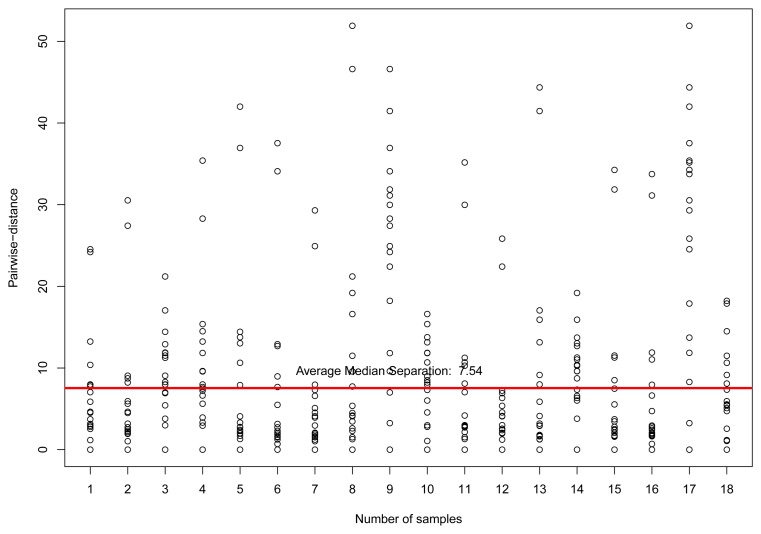
Pairwise-distances for temperature sensors in a range of 100 km from the coordinates of London, UK, in Dataset 2, considering only sensors with correlation *r* ≥ 0.8.

**Figure 12.**
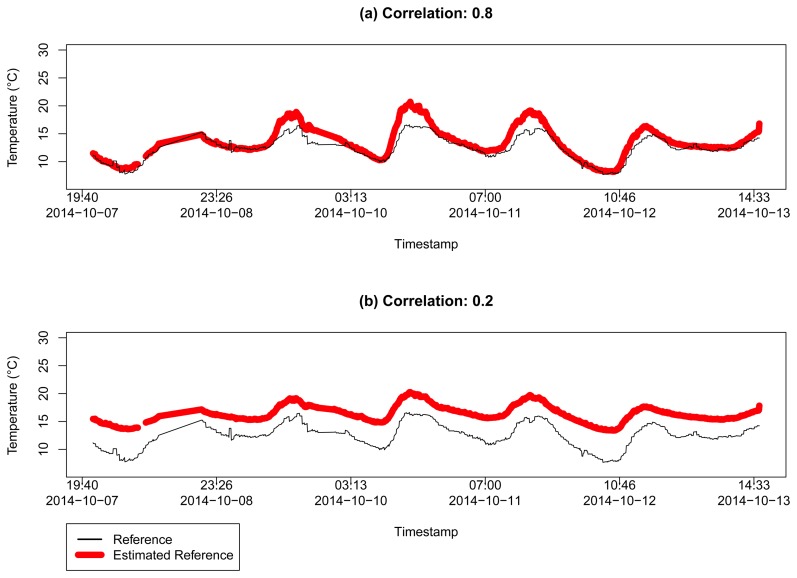
Estimation of the temperature *θ̂* for two different correlation limits in a range of 100 km from the coordinates of London, UK, in Dataset 2: (**a**) for sensors with correlation *r* ≥ 0.8 with MSE = 2.045454; and (**b**) for sensors with correlation *r* ≥ 0.2 with MSE = 15.63293.

For the temperature sensor selection, we considered the same steps and parameters of the previously mentioned experiment for Dataset 1. Figure 11 presents the resulting pairwise-distances for the 18 sensors found in Dataset 2. It is important to consider the time interval between collections, almost five months, to explain the difference in the number of detected sensors. In Figure 12, we have the estimation analysis for these reliable sensors. For different correlation limits, *r* ≥ 0.8 and *r* ≥ 0.2, we have the mean squared errors of 2.045454 and 15.63293, respectively, which are very close to the results in the previous experiment.

These results show that the proposed method is a reasonable approach to select reliable sensors, regardless of having a controlled set of sensors or not. Even when mixing the available sensors with different data classes, the selection of the reliable sensors gives a good approximation of the assumed correct temperature values.

For the next data classes, we considered the following parameters with Algorithm 1:

- For the pressure data, the correlation limit is defined as *r* ≥ 0.7, and the validity rule ensures that the average pressure of the sample data is upper and lower bounded by a factor of 4 from the average of the reference data.
- For the humidity data, we consider the correlation limit of *r* ≥ 0.65 and the validity rules consider a valid data to be a maximum humidity value of up to 100% and the minimum not less than 0%.
- For wind speed, even with a correlation limit of *r* ≥ 0.5, only one sensor was selected.

**Figure 13.**
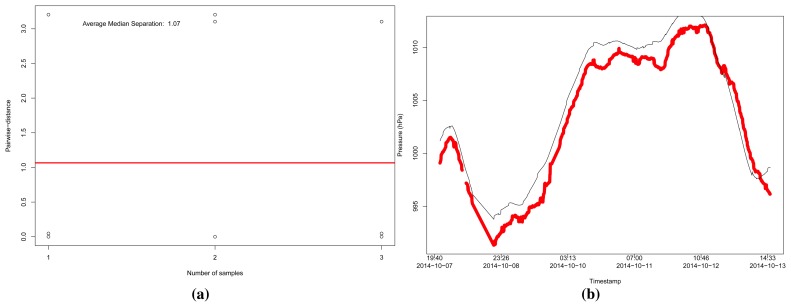
Analysis of pressure data from sensors in a range of 100 km from the coordinates of London, UK, in Dataset 2, considering a limit of *r* ≥ 0.7. (**a**) Pairwise-distances for pressure sensors; (**b**) Estimation of the pressure, resulting in a MSE = 2.66978.

**Figure 14.**
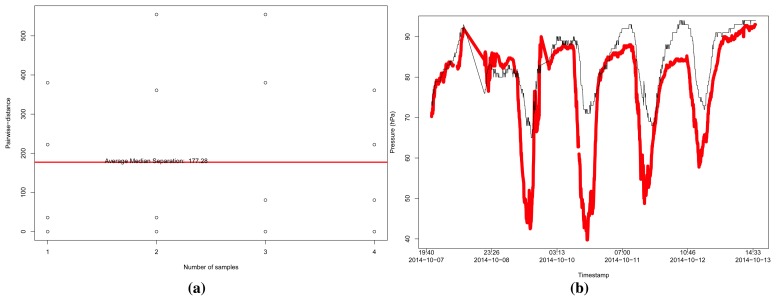
Analysis of humidity data from sensors in a range of 100 km from the coordinates of London, UK, in Dataset 2, considering a limit of *r* ≥ 0.65. (**a**) Pairwise-distances for humidity sensors; (**b**) Estimation of the humidity, resulting in a MSE = 104.213.

For the pressure data (Figure 13), we selected only three reliable sensors following our approach with *r* ≥ 0.7, but the refinement was able to combine those sensors and obtain a MSE of 2.66978. However, to the case of the humidity data (Figure 14), with a limit *r* ≥ 0.65, our approach was able to select only four reliable sensors, and the MSE was in the order of 104.213.

**Figure 15.**
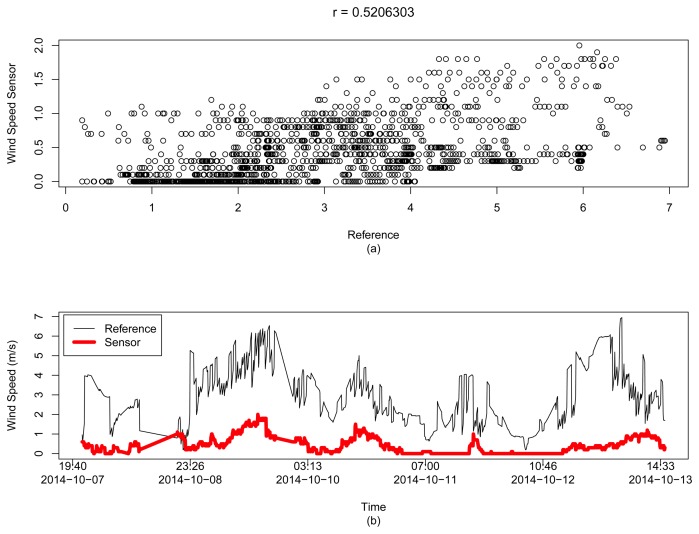
Analysis of the single wind speed sensor, with *r* = 0.5206303, in a range of 100 km from the coordinates of London, UK, in Dataset 2: (**a**) scatter plot between the reference and this sensor; and (**b**) the time series analysis of its readings.

For the wind speed data, Figure 15 shows the only sensor selected with a low correlation coefficient *r* = 0.5206303 between it and the reference data. For this case, there is no estimation of the correct value, and we must consider the readings of that sensor as our wind speed value.

In the experiments we conducted above for the second dataset, the number of selected sensors was significantly lower than the number of temperature sensors (Dataset 1). Thus, when the number of selected sensors, which provide the more reliable value, decreases, it is possible that there is no majority of sensors to converge to the expected output of the given environment data. Thereby, our approach depends on the number of sampled data, which we can not guarantee to occur. However, as stated earlier, in this era of IoT and big data, we can expect to have more and more data available in the future, and, if it is the case, makes our approach feasible.

In addition, on the majority of the selected sensors, the definition of the correlation limit value is crucial to the proper execution of our strategy. It is clear that the chosen correlation limit *r* directly impacts the number of selected sensors. As *r* approaches 1, the expected number of sensors that generate a data signal highly correlated to the reference value decreases and, conversely, this number is expected to increase when *r* approaches 0. However, it is also important to note that, even if the number of selected sensors increases, due to the choice of *r* ≈ 0, the quality of the sensor refinement algorithm will decrease, since it is expected that the mean squared error between the fused signals and the reference signal increases.

The trade-off between increasing the number of selected sensors and decreasing the MSE of their aggregation calls for a solution to determine the most appropriate correlation limit *r*. The optimum value of the correlation limits, for a given data class to be monitored, maximizes the number of selected sensors using the sensor selection strategy (Algorithm 1), and, once aggregated using the sensor refinement adjustments (Algorithm 2), results in the minimum mean squared error between them and the reference signal for this data class. In the case of our experiments, the choice of *r* was empirically determined by analyzing the selected sensors, and testing *r* in the reasonable range of 0.5 to 0.9.

## 5. Conclusions and Future Directions

Dealing with the uncertainties of the sensed data in a collaborative IoT scenario is a new and important problem that must be considered prior to the development of systems that can benefit from this data. The core of ubiquitous computing systems is based on the knowledge they infer about the state of the physical environment, and, thus, the reliability of the sensed data will directly impact the decisions and context-awareness of those systems.

In this work, we characterized the properties of the available sensed data from real deployed sensors in the current collaborative IoT for which we have a reference value. We conclude that, in order to safely use data available in the IoT, we need to perform a filtering process followed by a refinement of the selected data to increase its reliability. Thus, we proposed a simple and effective approach to select and refine the data from reliable sensors by a collaborative filtering technique.

Our assumption is that the combination of the readings for most of the reliable sensors converge to the correct value of the measured data. This hypothesis was validated through an experiment using data collected from real deployed sensors. The results show that the proposed method is a promising approach to select reliable sensors, regardless of having a controlled set of sensors or not. Even when mixing the available sensors with different data classes, the selection of the reliable data provides a good approximation of the assumed correct values, based on a reference sample.

When the number of selected sensors decreases, we may not have enough sensors and the collaborative filtering technique can not be expected to converge to the correct value of the given variable (in our example, environmental data). However, we can expect to have more sensors available in the future (possibly in the order of hundreds of millions), since we are entering a new era of computing technology with a very strong presence of the Internet of Things, and, thus, this issue will probably be minimized for different classes of sensor data.

As future work, we plan to extend our analysis of the properties of the sensed data in the IoT and its different classes, to better understand how the number of sensors impacts the collaborative filtering strategy, and infer about its precision limits. This analysis can help us to understand what is the minimum subset of available sensors in the collaborative IoT we need to assure the data quality.

In addition, an interesting point we plan to investigate is related to the heterogeneity and limitations of the IoT devices. Since IoT is comprised of different devices with different capabilities, the data accuracy provided by such elements can be compromised due to their limitations. Probably, the more limited the device is, the lower the data accuracy will be. Thus, we intend to study how these limitations can be used as another characteristic to be included in the sensor selection strategy.

It is also very important to investigate how the lack of a reference sensor influences the design of a solution based only on the analysis of the data itself. In that direction, we would like to study some possible strategies and the corresponding costs to model this as a mathematical/computational problem, such as an optimization problem, or even as a problem of statistical inference.

Finally, another direction is to explore the potential of ubiquitous computing by combining different data sources (e.g., humidity, rain precipitation, and even social networks) to improve the data reliability and accuracy.

## Figures and Tables

**Table 1.** Parameters for gathering data. Samples were collected from the region near London, UK, at different moments. The common parameters describe the intervals used for the data analysis.

**Common Parameters**	

**Location**	**London, UK**
Coordinates	lat. = 51.53, lon. = −0.10
Collection interval (min)	5
Range intervals (distance from coordinates in km)	10–1000
Reference weather service	Forecast.io

**Dataset 1**	

From	8 May 2014 14:05
To	12 May 2014 08:35
Total of samples	≈ 1070

**Dataset 2**	

From	7 October 2014 18:55
To	13 October 2014 12:40
Total of samples	≈1500
